# The rationale for druggability of CCDC6-tyrosine kinase fusions in lung cancer

**DOI:** 10.1186/s12943-018-0799-8

**Published:** 2018-02-19

**Authors:** Aniello Cerrato, Roberta Visconti, Angela Celetti

**Affiliations:** Institute for the Experimental Endocrinology and Oncology “Gaetano Salvatore”, Italian National Council of Research, Via S. Pansini 5, 80131 Naples, Italy

## Abstract

Gene fusions occur in up to 17% of solid tumours. Oncogenic kinases are often involved in such fusions. In lung cancer, almost 30% of patients carrying an activated oncogene show the fusion of a tyrosine kinase to an heterologous gene. Several genes are partner in the fusion with the three kinases ALK, ROS1 and RET in lung. The impaired function of the partner gene, in combination with the activation of the kinase, may alter the cell signaling and promote the cancer cell addiction to the oncogene. Moreover, the gene that is partner in the fusion to the kinase may affect the response to therapeutics and/or promote resistance in the cancer cells. Few genes are recurrent partners in tyrosine kinase fusions in lung cancer, including CCDC6, a recurrent partner in ROS1 and RET fusions, that can be selected as possible target for new strategies of combined therapy including TKi.

## TK fusions and their targeting in cancer

Structural chromosome rearrangements are frequent events in solid tumours and result in gene fusions, which can be diagnostic and prognostic for a selected tumour type. Current evidences indicate that the protein products of these fusions lead to a state of oncogene addiction, implying that they are ideal targets of anticancer drugs.

The list of known oncogenic fusions continues to grow with the improving of the detection methods, resulting to date in the identification of at least one gene fusion in up to 17% of solid tumours [1, 2]. Moreover, technical advances have simplified the detection of the gene fusions and in many cases they are now routinely searched for a more precise diagnosis and a more effective treatment of cancer patients.

One partner in such fusions is often an oncogenic tyrosine kinase (TK) that ends up to being constitutively active and with augmented, deregulated downstream signalling [3]. In most of the cases the heterologous gene fused to the kinase contributes the structural domains, which favour dimerization, trans-phosphorylation and constitutive activation of the kinase itself. However, upon the gene fusion, the active state of the kinase is promoted also by additional mechanisms, such as increased expression, altered turnover, conformational modifications, loss of the autoinhibitory domains and change of the substrates [1, 4–10].

Interestingly, the tyrosine kinase fusions to other genes also cause the inactivation of the heterologous partner gene as effect of its truncation, deletion or separation from its promoter [11]. Thus, independently from the activation of the kinase, the partner gene modifications might also provide additional effects on cellular signalling and gene transcription, including the activation of different pathways and chromatin remodelling [12]. Of note, in some cases, the gene that is partner in fusion with the TK might contribute, at least in part, to the oncogenic addiction and full cell transformation. Thus, different partner genes can differently affect tumour progression and response to therapeutics, including acquisition of resistance.

Tyrosine kinases are sensitive to different inhibitors (TKIs), generally classified in five types, depending on their mechanisms of action. Type I and II inhibitors occupy the adenosine trisphosphate (ATP)-binding pocket in both the active and inactive conformations of the kinase; type III and IV, by binding next to the ATP-binding site or outside of the cleft, act as allosteric inhibitors. Finally, type V inhibitors are referred as bivalent because they target simultaneously two distinct region of the protein kinase. Anyway, the majority of small molecules inhibit multiple kinases because the ATP-binding sites are highly conserved, leading to ‘off-target’ effects [13].

As kinases are ideal targets for therapy, several inhibitors are routinely used in the treatment of cancers harbouring TK activation, independently from the fact that its activation occurred upon point mutation or gene fusion. Unfortunately, the predicted efficacy of tyrosine kinase inhibitors based on biochemical, preclinical evidences obtained in vitro and in vivo, is not always confirmed in the clinic [14]. This divergence could be also ascribed to the fact that current TK inhibitors are more effective in patients harbouring TK point mutations than in patients carrying fusions of the same kinase [15]; thus, the need to deeply investigate the clinical impact of TK mutations versus TK fusion isoforms for their targeting. Owing to the low prevalence of the described molecular alterations, it is often impractical to clinically test the selected inhibitors in typical phase I/II trials, which only include patients with tumours of similar histology. Basket trials that involve patients with specific driver molecular alterations, regardless of the tumour histology, are therefore likely to provide more reliable results. Moreover, in the case of TK fusions, basket trials could also make possible to investigate the role of the different partner genes in the overall mechanisms of drugs sensitivity and/or resistance to TK inhibition, contributing additional clues for new strategies of combined therapy [16].

In this review we provide a speculative analysis of TK fusions, focusing on the paradigm of lung cancer, in order to supply useful information on drugs and drug cocktails that attack the unique networks activated by the fusions of the tyrosine kinases anaplastic lymphoma kinase (ALK), v-ros avian UR2 sarcoma virus oncogene homolog 1 (ROS1) and rearranged during transfection (RET), overall occurring in 5–10% of lung cancer. While this prevalence may seem low at first glance, the high incidence of lung cancer cases in the United States means that about 10,000 lung patients will be newly diagnosed with a rearranged TK in the next future [17, 18].

We also speculate on the role of CCDC6 as common partner of at least two TK, ROS1 and RET, for its targeting in combined therapies including TKIs in lung cancer treatment. Given the prevalence of CCDC6 fusion to ROS1 and RET, this analysis will identify and tailor a treatment, with the aim to enhance the TKI efficacy and to prevent resistance in almost 1000 lung cancer patients.

## The lung paradigm of TK fusions and their targeting

**Fig. 1:**
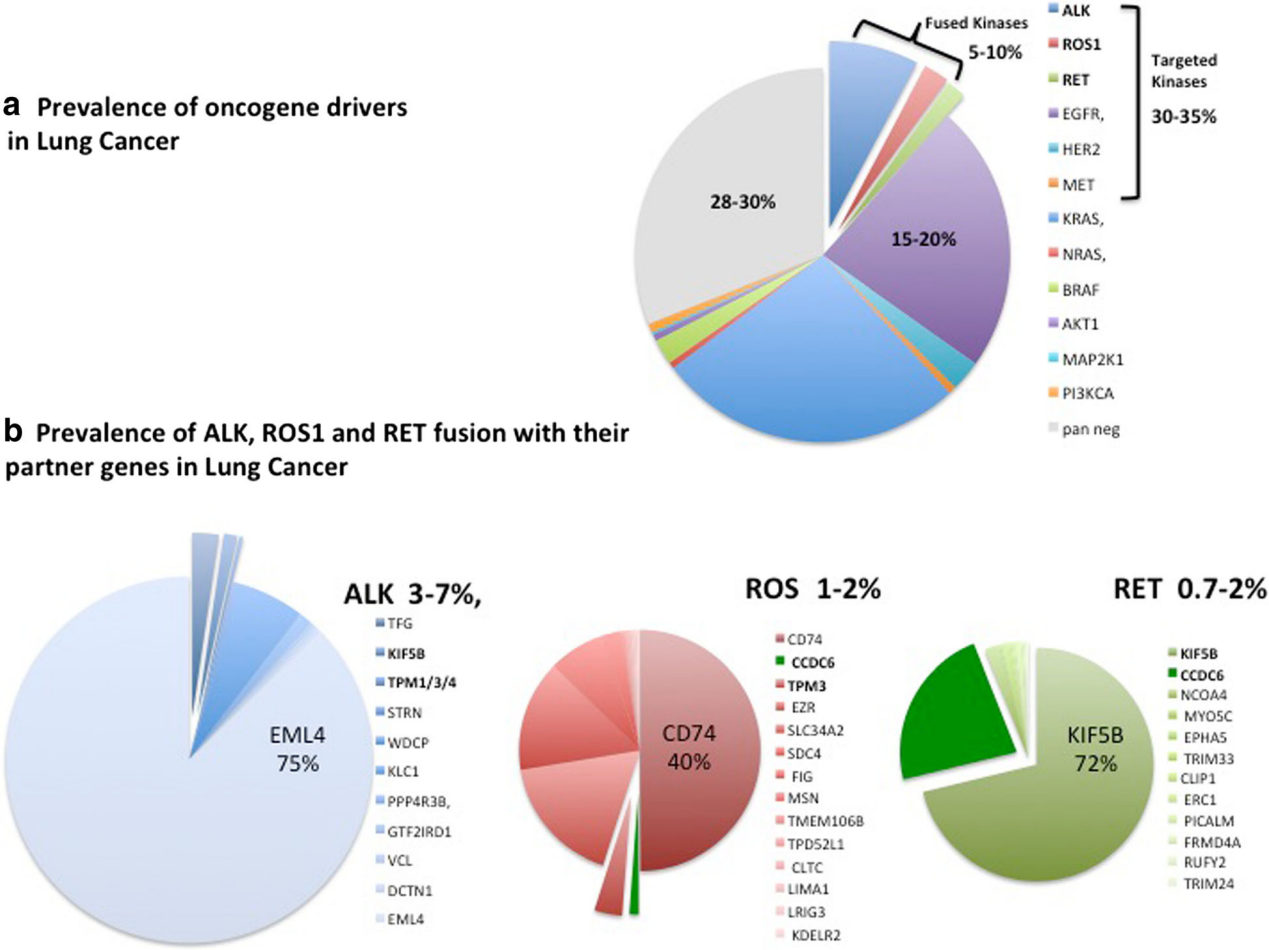
**a**) 70% of NSCLC show the activation of the indicated oncogene drivers while 30% are considered pan negative. Among the NSCLC carrying an oncogene driver, the identified alteration occurs in a tyrosine kinase (overall 30%–35% of NSCLC) that can be target of therapy. In a 30% of the NSCLC carrying an activated kinase (overall 5–10% of NSCLC) the activation is due to the fusion with a partner gene. **b**) Several genes are involved as partner in fusion to ALK, ROS1 and RET kinases in NSCLC. The impaired function of the partner gene may alter the cell signaling of the kinase and promote the cancer cell addiction to the oncogene. The involvement of the partner gene in NSCLC is related to its occurrence in the fusion with multiple kinases. Few genes, highlighted in bold, are recurrent partners in tyrosine kinase fusions in NSCLC. The common partner genes are sliced out of the large group of partner genes listed in the diagram

In lung, the somatic mutations of the EGFR gene are the best-characterized examples of oncogenic TK activation. Recently, however, TK fusions have been identified involving ALK, ROS1 and RET kinases in 3–7, 1–2, and 0.7–2% of lung cancer, respectively [16–18] (Fig. 1a).

The occurrence of the ALK, ROS1 and RET fusions seems to be mutually exclusive [19]. An early analysis of 1073 non-small cell lung cancer (NSCLC) specimens demonstrated no overlap between ROS1 and ALK rearrangements [20]. However, conflicting findings have subsequently been reported [21]. In the most recent and largest series to date, a total of 220 cases of ROS1-rearranged NSCLCs were examined. Amongst these tumours, ROS1 rearrangements never overlap with ALK fusions, and rarely co-occurr with oncogenic EGFR mutations (0.5%; 1/220) or KRAS mutations (1.8%; 4/220) [22, 23]. Thus, ROS1 rearrangements generally identify a unique molecular subset of NSCLC. Also the prevalence of the RET fusions greatly increases (from a median 1.8% to a significative 6.3%) when evaluated in 159 lung cancer patients wild type for EGFR, ALK, ROS1, BRAF, KRAS, HER2 [24–26].

Anaplastic lymphoma kinase (ALK), firstly identified as fused to nucleophosmin in an anaplastic large-cell lymphoma cell line, is normally expressed in the brain, small intestine, and testis [27]. ALK, that shows sequence similarity to the insulin receptor subfamily of transmembrane tyrosine kinases, is still considered an orphan receptor, even though pleiotrophin (PTN) and midkine (MDK), both secreted growth factors, are known to activate ALK downstream signalling [28, 29]. However, PTN and MDK effects on ALK activity are not due to direct binding [30]. Recently, it has been shown that heparin chains induce ALK dimerization, activation, and downstream signalling, indicating that heparin serves as ALK ligand or coligand [31]. While ALK gene amplification has been detected in a variety of tumours and gain-of-function mutations of ALK are described primarily in neuroblastoma, the most prevalent genomic ALK aberrations in human cancer are chromosomal rearrangements. ALK rearrangements have been found in multiple malignancies, including lung cancer, neuroblastoma, rhabdomyosarcoma, renal cell carcinoma, and inflammatory breast cancer [32].

ROS1 encodes a receptor tyrosine kinase (RTK) evolutionarily conserved in C. elegans, *D. melanogaster*, and vertebrates. However, the biological role of native ROS1 in humans has not yet been defined, and it remains an orphan RTK without a known ligand [17, 33]. ROS1 rearrangement has been firstly reported in an adult glioblastoma tumour [34]. Since the initial description, ROS1 fusions have been later detected in a wide range of malignancies including inflammatory myofibroblastic tumour [35, 36], cholangiocarcinoma [37], ovarian cancer [38], gastric cancer [39], colorectal cancer [40], angiosarcoma [41], spitzoid melanoma [42], and NSCLC [24, 43–56].

RET encodes a RTK whose primary ligands belong to the glial-derived neurotrophic factor (GDNF) family including GDNF, artemin, neurturin and persephin [57]. RET, specifically expressed on cells deriving from the neural crest, plays a key role mostly in organogenesis and development of the enteric nervous system [58, 59]. RET chromosomal rearrangements were initially identified in 5–40% of papillary thyroid cancers [18]. Furthermore, RET gain-of-function point mutations were also observed in up to 50% of sporadic medullary thyroid cancers [60] and the occurrence of germline mutations predispose patients to multiple endocrine neoplasia type 2 characterized by medullary thyroid cancer, pheochromocytoma and hyperparathyroidism [61]. Interestingly, RET somatic mutations were also described in small cell lung cancer patients [62–65] and cell lines [62, 66].

While ALK and ROS1 share a strong homology in the amino acidic sequence, with a 49% identity in the kinase domain and 77% identity in the ATP-binding site [44], ALK and RET share an homology of 37% in the amino acidic sequence of the kinase domain [67]. The identified homology provided the structural basis for a common targeting, at least for ALK and ROS1 [44]. Indeed, targeted therapies directed at constitutively activated oncogenic tyrosine kinases have proven to be remarkably effective against cancers carrying ALK and ROS1 fusions. In 3–7% of NSCLC patients who harbour the ALK fusions, the efficacy of the ALK directed TKI crizotinib have been reported in approximately 60% [68–70]. Crizotinib demonstrated remarkable efficacy, reminiscent of responses in ALK-rearranged patients, also in ROS1-rearranged NSCLCs, consequently gaining quick approval by the United States Food and Drug Administration as well as the European Medicines Agency in 2016. In fact, among 50 patients with ROS1-rearranged included in the phase I PROFILE 1001 study, crizotinib treatment resulted in an objective response rate (ORR) of 72%, with disease control rate (DCR) of 90% and a median progression-free survival (PFS) of 19.2 months [44]. On the other hand, disappointingly, early results obtained in patients carrying the RET rearrangements indicate a modest level of efficacy compared with the results obtained so far with inhibitors of ALK and/or ROS1. In the clinical setting, the RET patient benefit in terms of response (16% to 47%), and PFS (2 to 7 months), is clearly not comparable to that seen with other targeted agents in NSCLC patient bearing EGFR mutation (ORR: 56%–85%, median PFS: 9.2–13.7 months) [71], ALK (ORR: 60%–95%, median PFS: 8–11 months) [72] or ROS1 (ORR: 65%–85%, median PFS: 9.1–19.3 months) rearrangements [73].

All the kinases ALK, ROS1 and RET enhance cell proliferation and survival via activation of common downstream pathways RAS/MAPK, PI3K/AKT and JAK/STAT. However, extra-signalling pathways can be activated upon the TK fusions. The partner gene, besides affecting the stability of the fusion kinase, may drive the kinase activity on different substrates leading to the activation of additional signalling and to the alteration of the cellular metabolism [74–77]. Consequently, the switch-on/−off of numerous drivers and non-drivers genes confers cancer cells with “de novo” or acquired drug resistance. To this regard the partner gene fused to the tyrosine kinase may have specific role in the kinase activity as well as in the sensitivity of TK for its targeting. Several partner genes are fused to the mentioned kinases in lung. The most frequent derived-“driver oncogenes” are the echinoderm microtubule-associated protein-like 4 (EML4) fused to ALK, the kinesin family 5B (KIF-5B) fused to RET, and the cluster of differentiation 74 (CD74) fused to c-ros oncogene 1 (ROS1) which are closely involved in the therapeutic efficacy of each cognate targeting TKI [44, 78, 79] (Fig. 1a-b).

The EML4–ALK fusion occurs in 2–7% of NSCLC patients and is particularly prevalent in younger individuals, relative to those with wild-type NSCLC, and in never and/or light smokers (< 10 pack years) [24, 80, 81]. Following the discovery of this fusion in 2007, numerous additional ALK fusion partners have been identified, including TFG, KIF5B, KLC1, STRN, WDCP, TPM1/3/4, PPP4R3B, GTF2IRD1, VCL, and DCTN1 [1, 9] (Fig. 1b).

In the context of NSCLC, fusions involving ROS1 are described in 1–2% of patients [82]. A total of 14 different ROS1 fusion partner genes have been reported until now in lung cancer, including CD74 [1, 4, 24, 45–47], SLC34A2 [43–47], SDC4 [24, 44], EZR [24, 44, 48, 49], FIG. (46, 50), TPM3 [24, 44], LRIG3 [24], KDELR2 [46], CCDC6 [52], MSN [44, 53], TMEM106B [54], TPD52L1 [55], CLTC [56], and LIMA1 [4] (Fig. 1b). All ROS1 fusions retain the entire ROS1 kinase domain [24]. Similar to the ALK fusions, NSCLC patients with ROS1 fusions are typically younger than those with ROS1-wild-type and are never or light smokers [45].

Genomic screens have also identified at least 12 genes which are partner in fusion with RET in NSCLC: KIF5B [24–26, 79], CCDC6 [24], NCOA4 [83], MYO5C [84], EPHA5 [83], TRIM33 [85], CLIP1 [85], ERC1 [86], PICALM [83], FRMD4A [86], RUFY2 [87], and TRIM24 [87] (Fig. 1b).

The partner genes involved in the TK fusions in lung typically encode proteins that contain dimerization-competent motifs, suggesting that TK activation is mediated by dimerization and/or oligomerization. However, unlike the ALK and RET fusion, ROS1 fusions can signal as monomers, the mechanism underlying the constitutive activity of this kinase being still unknown [24]. In the case of tumours bearing tyrosine kinase rearrangement and treated with tyrosine kinase inhibitors, the fusion partner should be considered a potential target of combinatorial therapy to more potently block the aberrant activity of the fusion protein and eventually to overcome resistance.

In the case of RET fusions, the TK signals through, at least, some canonical RET signalling pathways [88], although the full downstream effects of RET fusions have not been fully explored [89]. In vitro evidences show that mitogen-activated protein kinase (MAPK) inhibitors have a stronger effect against medullary thyroid carcinoma (MTC) human cancer cell lines, carrying point mutated isoforms of the RET kinase, than against a papillary thyroid carcinoma (PTC) human cancer cell line, carrying the CCDC6-RET fusion [90].

Moreover, when assayed in flies, the fusions CCDC6-RET and NCOA4-RET exhibit differential sensitivity to clinically relevant kinase inhibitors, indicating that the signalling of RET fusion isoforms are at least in part dependent on the partner gene [14].

Accordingly, recent clinical studies suggest that different RET fusion variants might be differently sensitive to a specific TKI. In selected NSCLC, vandetanib (300 mg/day) was tested in a Japanese phase II study (LURET) including 17 RET-rearranged NSCLC patients, 31% of whom were CCDC6-RET-positive, 53% were KIF5B-RET-rearranged, 16% had an unknown RET status [91]. Treatment response and survival outcome were much higher in patients with the CCDC6-RET fusion subtype, with an 83% ORR and median PFS of 8.3 months, compared to 20% and 2.9 months, respectively, for patients with the KIF5B-RET fusion variant [91]. It is reasonable that, like in the case of the RET fusion isoforms, also the other TK fusions reported in lung (ALK and ROS1), acting through distinct pathways, might show different sensibility to selected tyrosine kinase inhibitors. To date, however, no definitive conclusions about a potentially diverse efficacy of anti-TK therapies according to different TK fusion variants have been achieved, mostly because the relative low frequency of TK rearrangements has allowed the analysis of only small subgroups, generating discordant data. Thus, the identification of the most efficient drug for each TK fusion isoform still remains a significant unmet need [92–94].

To this end, it is also urgent to deeper understand the mechanisms of drug resistance. Although the specific pathways and molecules involved will vary, the general principles of how biological mechanisms of resistance should be addressed are likely to be similar across cancer types.

The bedside-to-bench studies have advanced the understanding of the different biological mechanisms causing different types of resistance. For “on-target” resistance, for example, the strategy that seems mostly effective is to obtain new and more potent inhibitor compounds. Indeed, new agents in clinical development have demonstrated promising activity in crizotinib and TKIs type I resistant patients [95]. Now it is needed to investigate in detail the mechanisms of resistance related to the specific molecular alterations carried by the tumour and, thus, in our case, the drivers of and/or the mechanisms underlying the “de novo” and acquired resistance for ALK, ROS and RET fusion kinases [96]. These studies are, indeed, expected to identify the rationale for targeting parallel/compensatory signalling that could be preferentially activated in TK fusion with selected partners. A combined therapy seems to represent a valid alternative option for tumours with “de novo” or acquired resistance to TK activation. Thus, for selected isoforms of TK fusions it would be necessary to define the therapeutic synergism between different TK inhibitors, also in combination with new drugs intended to target the fusion partner.

## CCDC6/TK fusions and challenge of combined therapy to overcome resistance

Several genes have been identified as partners of TK fusions. Many of them are recurrent in fusions with different type of kinases, in different tumour types (Fig. 1b). The involvement of the same partner genes in different type of fusions might indicate that, besides the induction of the kinase activation, the partner gene might be functional to the full transformation and oncogene addiction of the cancer cells. The functional characterization of a partner gene frequently involved in TK fusions might help to select novel strategies for combined therapy in order to enhance the effects and/or overcome the resistance to the current treatments with TKIs.

In lung, four genes have been identified as recurrent partners in fusion with at least two of the three kinases rearranged. TPM-family genes and TFG (TRK-fused gene) have been found fused to ALK and ROS1 kinases in overall 5% of cases among those carrying a TK fusions; KIF5B is rearranged with both ALK and RET kinases in almost 70% of cases carrying a TK fusions, and CCDC6 is a common partner of ROS1 and RET kinases in about 25% of cases [9, 15, 19] (Fig. 1b).

CCDC6 (coiled coil domain containing 6) is a ubiquitously expressed 65 kDa nuclear and cytosolic protein phosphorylated by S/T kinases, that exerts a negative regulation of the CREB1 transcriptional activity, in a SUMO2-regulated manner [97–101]. CCDC6 is involved in apoptosis, while its truncated mutant of 1-101aa, which corresponds to the portion of CCDC6 included in the CCDC6-RET fusion, acts as dominant negative on CCDC6 nuclear localization and apoptotic function [97]. CCDC6 is an ATM substrate at T434 and is involved in ATM-mediated cellular-response [98]. Following genotoxic stress, CCDC6 interacts with PP4c and negatively modulates the phosphatase enzymatic activity toward the dephosphorylation on S139 of the histone H2AX (γH2AX), the specific marker and efficient coordinator of the DNA repairing process. Thus, in CCDC6 depleted cells, the loss or inactivation of CCDC6 accelerates the dephosphorylation of γH2AX, resulting in defective checkpoint activation, defective G2 arrest and premature mitotic entry. Moreover, CCDC6 depleted cells affects the γH2AX foci formation and favors the repair of the DNA DSBs repair by non-homologous-end-joining more prone to errors than homologous recombination, in a shorter time compared to controls [99]. Of notice, the tumor suppressor DNA repair function of CCDC6 has been found lost, by several mechanisms, in many human cancer [102].

**Fig. 2:**
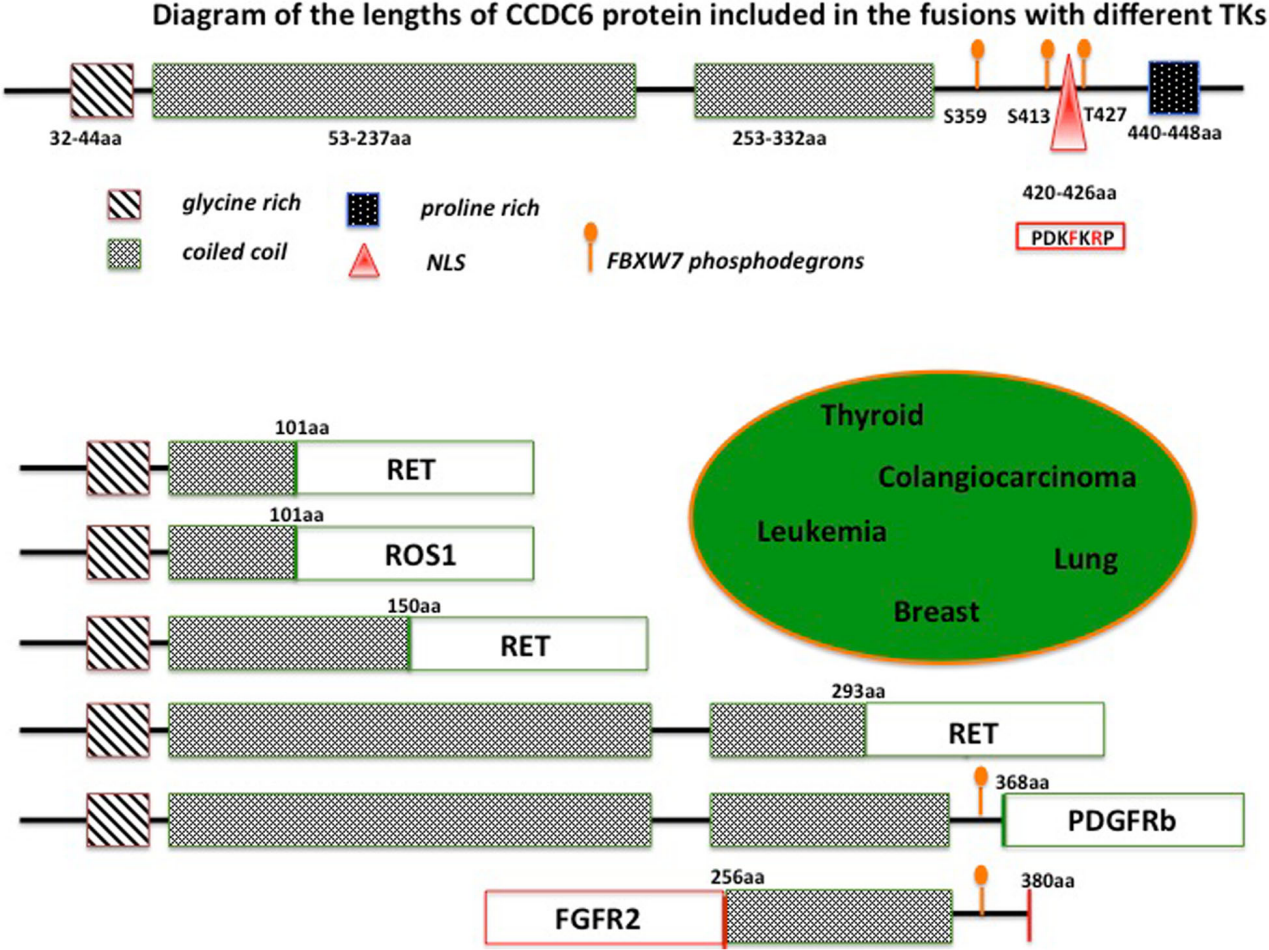
Diagram of the full length of CCDC6 protein (upper part of the figure) and its portions fused to the different tyrosine kinases (RET, ROS1, PDGFRb, and FGFR2) in several tumor types grouped in the elipse (lower part of the figure). The protein regions contributed by CCDC6 to the chimeric oncogenes always include the coiled-coil domain and lack the nuclear localization peptide (NLS). In almost all the chimeric oncogenes the length of CCDC6 protein does not include the FBXW7 phosphodegron (aa S359, S413, T427) which are known to be relevant for the CCDC6 protein stability

CCDC6 has been involved in different rearrangements with several tyrosine kinases (RET, PDGFRb, ROS1, FGFR2) in several tumours (thyroid, lung, leukemia, breast, iCCA) [102] (Fig. 2).

In CCDC6-TK fusions, CCDC6 contributes with portions of different length of its protein [102] (Fig. 2). When rearranged with RET, CCDC6 contributes aminoterminus portions corresponding to 101aa, identified in most of the cases of lung and thyroid tumours, or portions corresponding to 150aa or 293aa, reported at very low percentage in thyroid tumours [24, 103–105]. In leukemia, CCDC6 rearranged with PDGFRβ contributes an aminoterminus portion of 368 aminoacids [106]. In breast and iCCA, a FGFR2-CCDC6 fusion has been recently identified and the CCDC6 gene contributes the coiled coil region at the 3′ terminus of the chimeric oncogenes [6, 107] (Fig. 2).

It will be important to evaluate whether the different lenghts of the CCDC6 portion included in the fusions could influence the stability of the oncogenic kinase and its sensitivity to different drugs. Accordingly, strong evidences suggest that the ALK tyrosine kinase activity is not the only driving force for oncogenesis in EML4-ALK positive NSCLC. In case of EML4-ALK fusions, there are many evidences suggesting that the specific targeting of EML4 can be a rewarding strategy to avoid resistance. Fifteen different EML4-ALK variants contain the entire ALK kinase domain but differ in the point of fusion with the EML4 gene including the EML4 TD domain required for oligomerization of the fusion proteins but diverging in the length of the EML4 TAPE domain present [108–111]. In vitro studies have demonstrated that the length of the TAPE domain greatly influences fusion protein stability: the EML4-ALK variants 1 and 2, expressing a partial TAPE domain, are more unstable than variant 3a/b that lacks the TAPE domain. Remarkably, this has enormous clinical impact, as the more stable variants are less sensitive to ALK inhibitors as demonstrated in preclinical studies and by retrospective studies of NSCLC patients expressing different fusion variants and treated with crizotinib [112, 113]. Thus, the different length of the EML-4 TAPE domain in EML4-ALK variants, determining the stability of the fusion proteins, has relevant biochemical consequences and clinical implications [112, 113].

Several evidence suggest that most of the oncogenic kinases rely on chaperone proteins, such as HSP90, for stability. The 17-DMAG HSP90 inhibitor has been tested in Ba/F3 cells expressing different EML4-ALK variants. Remarkably, sensitivity to ALK kinase inhibition did not correlate with sensitivity to 17-DMAG. Moreover, the combination of crizotinib and 17-DMAG induced synergistic cytotoxicity in all ALK fusion–expressing cells, with the maximal synergistic cytotoxicity observed in cells expressing the most stable EML4-ALK variant 3a [112].

CCDC6 stability and turnover are highly regulated by post-translational modifications following the phosphodegron recognition (Fig. 2) by the E3-Ubiquitin ligase, FBXW7, and upon the activity of the de-ubiquitinase enzyme, USP7 [114]. The targeting of USP7 to reduce CCDC6 levels appears to be useful for the establishment of new therapeutic approaches in cancer treatment [115–117], since it could also affect the stability and turnover of the TKs fused to CCDC6. Combined effects of the inhibitors of USP7 and TKI should be investigated in lung tumours carrying the CCDC6/TK fusions.

In addition to the hypothetical role of CCDC6 on the TK stability and on the development of “on-target” resistance, the impairment of CCDC6 in tumours carrying the fusions may induce “off targets” effects as well as “off targets” resistance. It has been recently postulated that the CCDC6 impairment derived from its truncation in fusion oncogenes, enhances tumour progression and impacts on selective response to therapeutics offering new chances for a tailored therapy and novel challenges to overcome drug resistance [118].

**Fig. 3:**
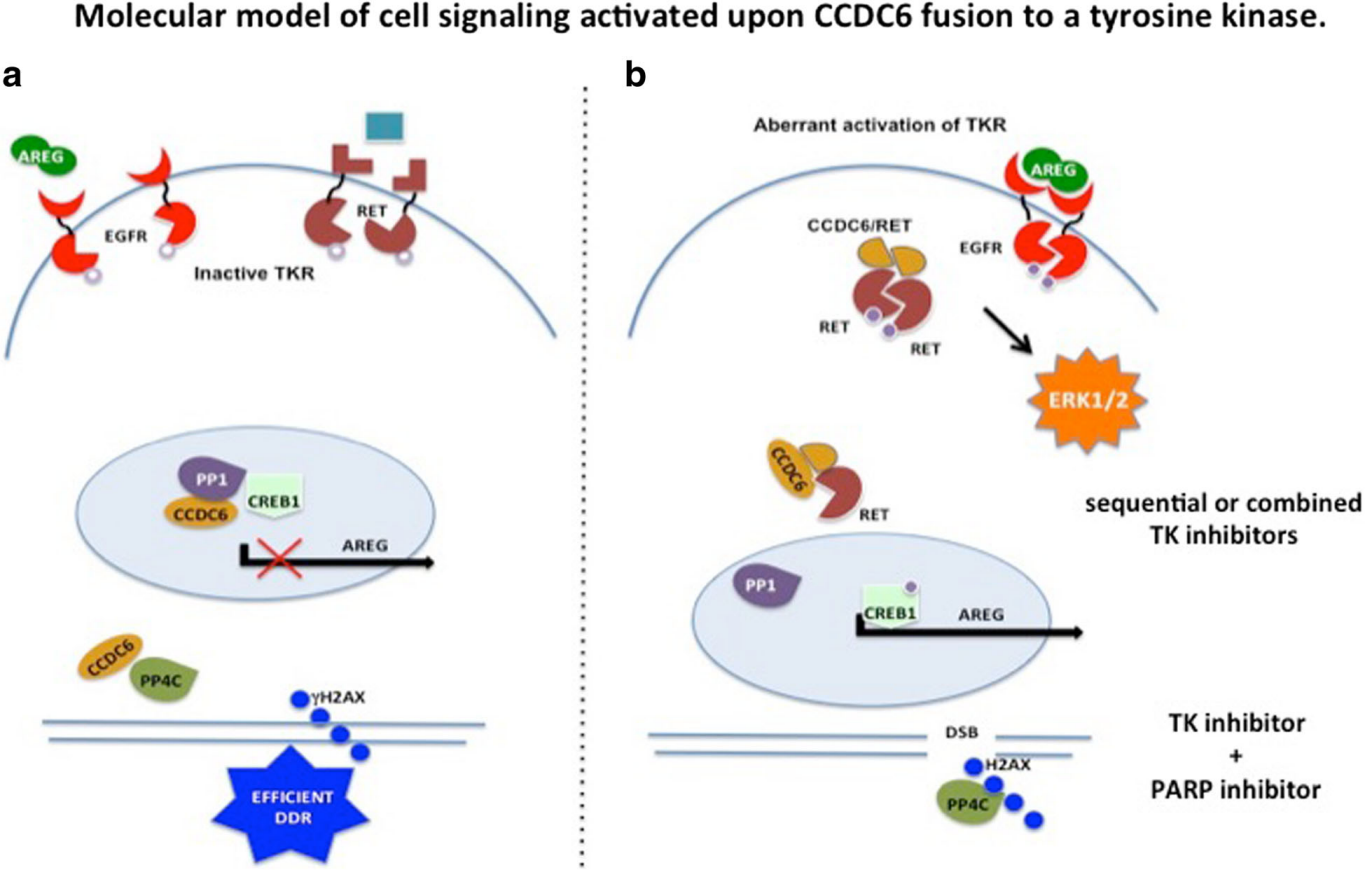
Molecular model of cell signaling in CCDC6 unperturbed condition (**a**) and upon CCDC6 fusion to the tyrosine kinase (ie RET) (**b**). **a**) In CCDC6 unperturbed condition. i) CCDC6 can complex with CREB1 and the phosphatase PP1, keeping CREB1 in an inactive state for the transcription of Amphiregulin (AREG). ii) CCDC6 can promote the maintenance of phosphorylated H2AX, on S139, in the foci of DNA Damage Repair (DDR) by holding back the histone H2AX specific PP4C phophatase, allowing the correct DNA repair process. **b**) Upon CCDC6 fusion to the kinase (ie RET). i) the chimeric oncogene CCDC6/TK forms homodimers which activate the MAPK/ERK cascade. ii) the chimeric oncogene CCDC6/TK forms heterodimers with the wild-type CCDC6 protein which act as dominant negative on CCDC6 nuclear localization and function. In this condition CCDC6, unable to repress CREB1 activity, results in an autocrine loop of AREG for the autonomous activation of EGFR and MAPK/ERK cascade. Moreover, in this condition CCDC6 is unable to hold back the PP4C phosphatase directed towards the de-phosphorylation on S139 of the histone H2AX in response to DNA damage (DDR), leading to an uncorrect repair of the Double Strand Breaks (DSBs) in the TK activated cancer cells. On these bases, hypothetical innovative therapeutic approach are suggested

CCDC6 has been identified as a negative regulator of CREB1 dependent transcription and tumours harboring the CCDC6-RET oncogene or occurring in Ccdc6-ex2 knock-in mice exhibit an enhanced phosphorylation and activity of CREB1 with a consequent increased expression of Amphiregulin (AREG) one of the known ligand for the EGFR family [100, 119, 120]. The autocrine production of Amphiregulin (AREG) is a parallel compensatory survival signaling responsible of “off-targets” TKIs resistance identified in different tumour types. Moreover, recent findings have shown that EGFR signaling can provide a critical adaptive survival mechanism that allows cancer cells to evade kinase fusion specific inhibitors, providing a rationale to co-target EGFR in order to reduce risks of developing drug resistance [121]. If this molecular mechanism will prove to be crucial for cancer cell proliferation, the absence of CCDC6 may have important therapeutic effects for the targeting of the EGFR family members in determined tumours [122], (Fig. 3).

Interestingly, the activation of epidermal growth factor (EGF) is known to trigger resistance to RET inhibitors, bypassing survival signaling through ERK and AKT activation. Targeting EGFR using specific TKIs like gefitinib brings back cancer cells sensitivity to RET inhibitors [120]. Furthermore, the inhibition of the EGF signaling by EGFR small interfering RNA (siRNA), anti-EGFR antibody (cetuximab), and EGFR-TKi (Iressa) determined an increase in the sensitivity to RET inhibitors in lung cancer cells carrying CCDC6-RET fusion [123] (Fig. 3). Thus, if a role can be ascribed to CCDC6 in the activation of the EGFR signaling, the detection of CCDC6 impairment upon TK fusion could represent an indication for targeting EGFR in cancer therapy (Cerrato A, Di Domenico I, Morra F, Celetti A, manuscript in preparation). Therefore a sequential or combined treatment scheme by small molecules inhibitors can be envisaged (Fig. 3).

In both preclinical and clinical studies, evidences suggest that CCDC6-RET fusion is selectively responsive to vandetanib compared to NCOA4-RET and KIF5B-RET fusions. This finding may support the fact that RET fusions involving CCDC6 are more sensitive to TK inhibitors that can inhibit also the EGFR signaling as “off target” effect [14, 102].

Interestingly, the activation of CCDC6-RET has been identified in post progression samples of lung cancer patients, which developed resistance to EGFR TKIs [124]. Thus, the inactivation of CCDC6 in combination with the activation of RET could be identified as additional mechanism of EGFR TKIs resistance, beside the EGFR T790 M mutation, EGFR amplification, HER2 amplification, MET amplification, PIK3CA mutation, BRAF mutation [125, 126].

On these bases, TKIs that show “off targets” effects like vandetanib might be more effective in those cases in which RET TK activation is combined with the enhancement of EGFR signaling because of the CCDC6 impairment. In particular, “off targets” effects of TKIs could be considered a better therapeutic option in thyroid or lung tumours that carry CCDC6/RET fusion protein, in order to avoid tumour progression and TKIs resistance (Fig. 3). Moreover, loss or inactivation of CCDC6 in cancers, by accelerating the dephosphorylation of the histone γH2AX results in defective G2 arrest and premature mitotic entry [99]. This suggests that tumours with defective CCDC6 signaling could be sensitive to the combination of DNA-damaging and anti-mitotic drugs [127–129].

Preclinical studies indicate that the attenuation of CCDC6 in cancer confers resistance to cisplatinum and sensitizes the cancer cells to the small molecule inhibitors of Poly (ADP-ribose) polymerase (PARP1/2) in accordance with its role in the DNA damage response [91, 130–132]. The important role of CCDC6 in the DNA damage could impact genome stability in primary tumours [133], as reported also for other genes whose products participate in DDR and are commonly deregulated or inactivated in tumors [134]. CCDC6 impairment upon fusions, altering the DDR process in TK addicted cells, might promote the tumour heterogeneity by enhancing ongoing error-prone DNA replication with differential selection pressures and drug sensitivity [135] (Fig. 3). Thus, CCDC6, when fused to ROS1 and RET kinase, could be imagined as a predictive biomarker of resistance to conventional single mode therapy and provides indications about the tumour sensitivity to PARPi in combination with TKI in NSCLC. A combination therapy of TKIs and PARPi should be valuable in lung or different cancer types that result defective for CCDC6 because of qualitative targeting of tumour over non-tumour cells (Fig. 3).

## Conclusion

In conclusions, it is plausible to envision a future in which the different TK fusion variants of CCDC6-ROS1 and CCDC6-RET will be used as biomarkers of sensitivity to therapy with TK inhibitors in combination with drugs targeting protein stability, such as chaperone, proteasome, and deubiquitinase inhibitors.

Moreover, in cancers carrying kinase fusion and actionable partners, an improved final outcome of the disease should be attained when a strategy of combined therapy is utilized, with a reduction of the side effects due to the conventional therapy. For example, the combined treatment with diverse TKIs, including gefitinib, should target preferentially the tumour cells carrying CCDC6 rearrangements, while normal cells should be more resistant to the combination of these two drugs.

Tumours carrying the CCDC6 fusion might be prone to the tumour heterogeneity because of error-prone DNA replication leading to a different selection pressure upon TK targeting. Thus, patients carrying CCDC6/TK fusion may benefit of a combined therapy of TKI and PARPi in order to avoid selection of TKI resistant cancer cells.
